# Role of Cone Beam Computed Tomography in Evaluation of Radicular Cyst mimicking Dentigerous Cyst in a 7-year-old Child: A Case Report and Literature Review

**DOI:** 10.5005/jp-journals-10005-1438

**Published:** 2017-06-01

**Authors:** BS Mahesh, Shilpa P Shastry, Padmashree S Murthy, TR Jyotsna

**Affiliations:** 1 Senior Lecturer, Department of Oral Medicine and Radiology, Vydehi Institute of Dental Sciences, Bengaluru, Karnataka, India; 2 Senior Lecturer, Department of Oral Medicine and Radiology, Vydehi Institute of Dental Sciences, Bengaluru, Karnataka, India; 3 Professor and Head, Department of Oral Medicine and Radiology, Vydehi Institute of Dental Sciences, Bengaluru, Karnataka, India; 4 Postgraduate Student, Department of Oral Medicine and Radiology, Vydehi Institute of Dental Sciences, Bengaluru, Karnataka, India

**Keywords:** Cyst, Deciduous dentition, Dentigerous cyst, Radicular cyst.

## Abstract

**Aim:**

To report a rare case of large radicular cyst-associated deciduous tooth and to discuss the importance of cone beam computed tomography (CBCT) in diagnosing the condition.

**Background:**

Radicular cyst is the most common cyst affecting the permanent teeth, but its occurrence in deciduous teeth is rare. Most of the radicular cysts are asymptomatic and are discovered accidentally when radiographs are taken. Conventional radiographs show two-dimensional images of three-dimensional objects. Cone beam computed tomography provides undistorted three-dimensional information of hard tissues and gives adequate spatial resolution.

**Case report:**

A 7-year-old child, with a complaint of swelling in the maxillary anterior region, was diagnosed with radicular cyst in relation to primary maxillary right central incisor based on CBCT and histopathological features.

**Conclusion and clinical significance:**

Early diagnosis and prompt treatment of radicular cyst in primary dentition is important to prevent damage to permanent tooth.

**How to cite this article:**

Mahesh BS, Shastry SP, Murthy PS, Jyotsna TR. Role of Cone Beam Computed Tomography in Evaluation of Radicular Cyst mimicking Dentigerous Cyst in a 7-year-old Child: A Case Report and Literature Review. Int J Clin Pediatr Dent 2017;10(2):213-216.

## INTRODUCTION

Radicular cyst is the most common cyst of the jaws, with frequency of about 7 to 54% in permanent dentition. But radicular cyst of deciduous dentition is extremely rare (0.5 to 3.3%).^1^ Radicular cyst arises from the epithelial residues in the periodontal ligament as a result of inflammation, following the death of the dental pulp. These cysts usually involve the apex of the teeth.^2^ Most of the radicular cysts are asymptomatic and are discovered accidentally when periapical radiographs are taken. Radiographically, radicular cyst appears as a well-defined round or oval unilocular radiolucency with radiopaque sclerotic margin in the periapical region of involved tooth, but in case of an infected cyst, the radiopaque margin disappears because of rapid growth of the cyst.

The conventional radiographs show two-dimensional image of the three-dimensional object and its surrounding structures.^3^ Cone beam computed tomography (CBCT) provides three-dimensional images of the object from sagittal, coronal, and axial direction, to overcome the defects of two-dimensional image, such as overlap and deformation.^4^ Hence, the aim of this study is to report a rare case of a large radicular cyst associated with maxillary right primary central incisor and to discuss the importance of CBCT in diagnosing the condition.

## CASE REPORT

**Fig. 1:**
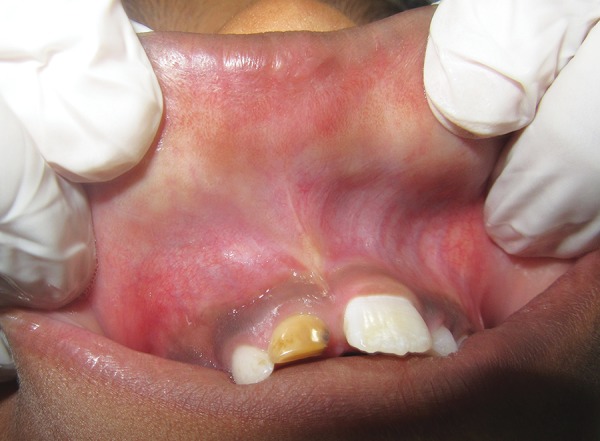
Swelling in the right maxillary anterior tooth region

**Fig. 2:**
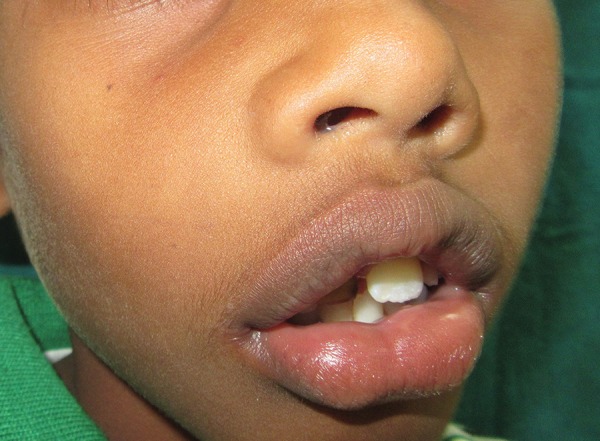
Intraoral view showing vestibular swelling, discolored, and proximal caries in relation to 51

A 7-year-old male patient reported to the Department of Oral Medicine and Radiology with the chief complaint of swelling in the upper front tooth region since 25 days. Swelling was small initially, which gradually increased to the present size. Swelling was associated with pain, which was continuous, dull, and mild in nature. Patient gave history of trauma to the upper front tooth region 2 years back. There was no history of pus discharge and ulceration associated with the swelling. On examination, extraorally diffuse swelling in the right maxillary anterior region in the middle third of the face was noted with upward displacement of nares (Fig. 1). The swelling was tender on palpation, hard in consistency with no rise in local temperature. Intraorally swelling extended from labial frenum to distal aspect of maxillary right first deciduous molar (tooth number 54) with obliteration of labial and buccal sulcus. Discoloration and proximal caries was seen in relation to maxillary right deciduous central incisor (tooth number 51). Also, maxillary right permanent central incisor (tooth number 11) was clinically missing and maxillary left permanent central incisor (tooth number 21) was erupted (Fig. 2). Upon palpation, intraorally swelling was hard in consistency and tender. Mobility in relation to 51 and 52 was also observed. Based on the history and clinical findings, a provisional diagnosis of radicular cyst of 51 and a differential diagnosis of dentigerous cyst in relation to clinically missing 11 was given.

**Fig. 3:**
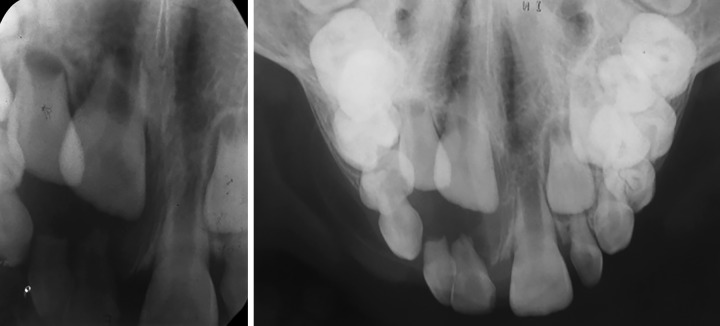
Intraoral periapical and occlusal radiograph

Patient was then subjected for radiographic investigation. Intraoral periapical (IOPA) radiograph and occlusal radiograph revealed proximal caries involving enamel and dentin without pulpal involvement in relation to 51, well-defined radiolucency in the periapical regions of 51 and 52, and this radiolucency was enveloping the coronal portion of 11 and 12. Root resorption of 51 and 52 were noted. Occlusal radiograph also revealed the presence of buccal cortical plate expansion (Fig. 3).

**Fig. 4:**
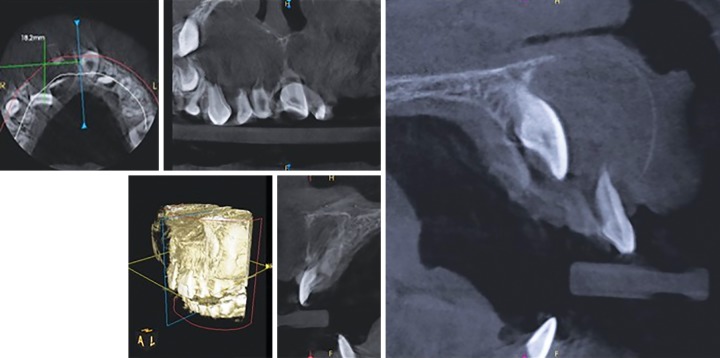
Cone beam computed tomography showing radicular cyst of 51, with apical displacement of 11

**Fig. 5:**
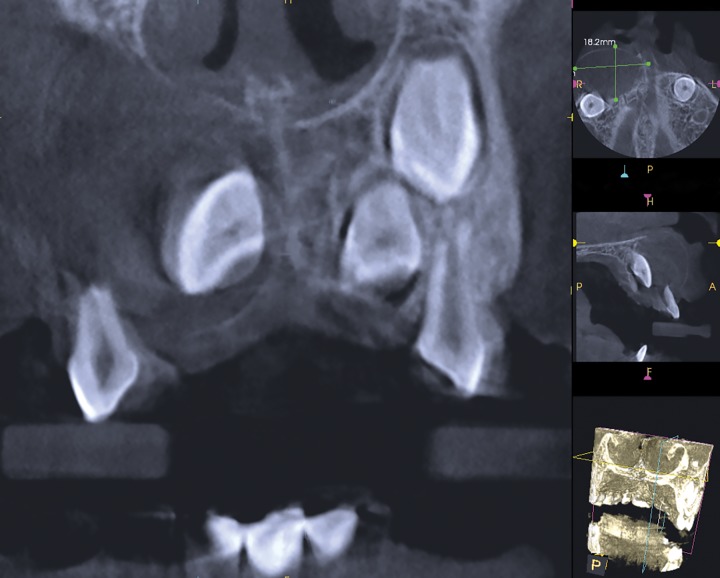
Cone beam computed tomography showing the radicular cyst causing thinning of the nasal floor

Cone beam computed tomography revealed osteolytic lesion with sclerotic margin in the periapical region of 51, measuring about 1.8 × 2.5 × 2.5 cm in dimension. The tooth 11 was displaced apically, root resorption of 51 and 52 was seen. Thinning of nasal floor with buccal cortical plate expansion was present (Figs 4 and 5). Based on CBCT features, radiographic diagnosis of radicular cyst of 51 was given. Cyst was enucleated with extraction of 51 and 52 and preservation of 11. Histopathologically the specimen revealed cystic epithelium and fibrous connective tissue capsule. The epithelium showed arcad-ing pattern, with dense collagen fibers in the connective tissue. These histopathological features confirmed the diagnosis of radicular cyst in relation to 51.

## DISCUSSION

The incidence of radicular cyst in deciduous tooth seems to be low as compared with permanent tooth. The probable reasons could be the shorter lifespan of primary teeth, and presence of numerous accessory canals leading to easy drainage in the primary teeth. Further, extraction or exfoliation of the associated deciduous tooth often leads to resolution of lesion on its own.^5^ Commonly, cyst is associated with carious, nonvital, discolored, or fractured tooth or teeth. However, in primary teeth, Grundy, Adkins, and Savage reported that teeth with endodontic treatment are more prone for development of radicular cyst. It was proposed that antigen stimulation by various products used for the endodontic treatment might be associated with development of a radicular cyst in primary teeth.^6^ Trauma can also be less commonly attributed etiology of development of radicular cyst in primary teeth.^5^

Radicular cysts of deciduous teeth occur in the age group of 3 to 19 years, with male predominance. Most commonly reported teeth are mandibular molars (67%), maxillary molars (17%) followed by anterior teeth.^7^ Present case was reported in a 7-year-old male patient, involving maxillary primary incisor following trauma to the upper anterior teeth.

Radicular cysts associated with primary teeth may cause expansion of cortical plates, especially buccal cortical plate.^8^ Radicular cysts may also cause resorption, delayed eruption, malposition enamel defects, or damage to the developing permanent teeth.^9^

Radiographic changes of radicular cyst include well-defined radiolucency, expansion of cortical plates with thin reactive cortex, and displacement of permanent tooth. In the present case, IOPA and occlusal radiograph showed well-defined radiolucency in the periapical regions of 51 and 52, and this radiolucency was enveloping coronal portion of 11. But distinguishing whether this radiolucency was associated with 51 or 11 was difficult to determine. Cone beam computed tomography gave us the clear evidence of well-defined radiolucency, in the periapical region of 51, while the tooth 11 was displaced apically. This radiolucency was not attached to the cervical aspect of 11. Shear and Speight^2^ reported that the characteristic radiographic feature of dentigerous cyst is that pericoronal radiolucency is attached to cementoenamel junction of the associated teeth. Cone beam computed tomography clearly revealed that radiolucency was not attached at the cementoenamel junction of central incisor, but enveloping the periapical region of deciduous teeth. Thus, preopera-tive diagnosis of radicular cyst was substantiated. Further, during surgical procedure, the cyst was associated with deciduous tooth and permanent central incisor was pushed apically. Moreover, on histopathological examination, diagnosis of radicular cyst was confirmed.

Distinguishing between radicular cyst and dentiger-ous cyst is very essential as the treatment plan for both would be different. Cone beam computed tomography gives adequate three-dimensional views of oral and maxillofacial structures. In the present case, CBCT assisted in the preoperative diagnosis of radicular cyst, thus helping in the surgical planning.

Hill^10^ reported growth of 4 mm each year for radicular cyst, but in our case growth rate was faster. Treatment of radicular cyst in deciduous teeth involves complete enucleation of the cyst and preservation of the permanent successor teeth. Marsupialization is a conservative intervention and is done for larger cysts. But, with this technique, pathologic tissue is left *in situ* and multiple visits are required for regular draining of the cavity.^11^ In our case, extraction of deciduous teeth, cyst enucleation, and preservation of permanent teeth were done.

## CONCLUSION

Considering the low incidence of radicular cysts in primary dentition, this case report informs possibility of a large radicular cyst secondary to trauma in maxillary anterior deciduous teeth and highlights the significance of CBCT in aiding the diagnosis of the condition because of adequate spatial resolution and undistorted hard tissue information. Early diagnosis and prompt treatment of radicular cyst in primary dentition is important to prevent damage to permanent tooth as well as the need for prevention of invasive surgical treatment.
